# Frequency chasing of individual megadalton ions in an Orbitrap analyser improves precision of analysis in single-molecule mass spectrometry

**DOI:** 10.1038/s41557-022-00897-1

**Published:** 2022-03-10

**Authors:** Tobias P. Wörner, Konstantin Aizikov, Joost Snijder, Kyle L. Fort, Alexander A. Makarov, Albert J. R. Heck

**Affiliations:** 1 Biomolecular Mass Spectrometry and Proteomics, Bijvoet Center for Biomolecular Research and Utrecht Institute for Pharmaceutical Sciences, University of Utrecht, Utrecht, the Netherlands; 2 Netherlands Proteomics Center, Utrecht, the Netherlands; 3 Thermo Fisher Scientific, Bremen, Germany

**Keywords:** Mass spectrometry, Single-molecule biophysics

## Abstract

To enhance the performance of charge-detection mass spectrometry, we investigated the behaviour of macromolecular single ions on their paths towards and within the Orbitrap analyser. Ions with a mass beyond one megadalton reach a plateau of stability and can be successfully trapped for seconds, travelling a path length of multiple kilometres, thereby enabling precise mass analysis with an effective resolution of greater than 100,000 at a mass-to-charge ratio of 35,000. Through monitoring the frequency of individual ions, we show that these high-mass ions, rather than being lost from the trap, can gradually lose residual solvent molecules and, in rare cases, a single elementary charge. We also demonstrate that the frequency drift of single ions due to desolvation and charge stripping can be corrected, which improves the effective ion sampling 23-fold and gives a twofold improvement in mass precision and resolution.

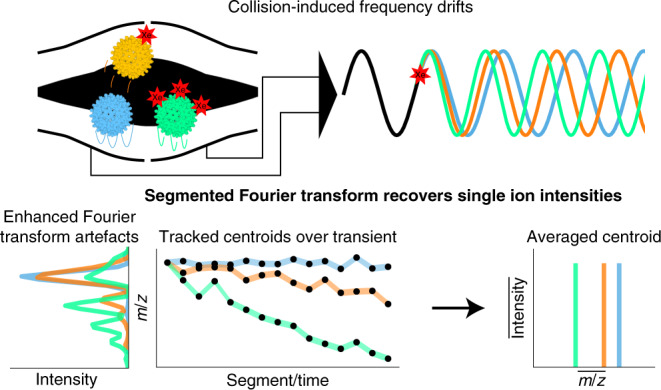

## Abstract (web summary)

The mass precision and resolution in charge-detection mass spectrometry can be improved by correcting frequency drifts of single ions. Now, chasing these individual ions for seconds in an Orbitrap mass spectrometer has revealed the exceptional stability of ultra-high-mass ions, culminating in an effective resolution of greater than 100,000 at *m*/*z* = 35,000.

## Main

Mass spectrometry (MS) offers a versatile analytical approach to characterize molecules with high precision and throughput and can be applied to molecules with a mass ranging from a few to several million dalton (Da). For analysis of molecules at the higher end of this mass range (for example, macromolecular assemblies), electrospray ionization under non-denaturing conditions (‘native MS’^1^) is the method of choice to make such ‘elephants fly’^2^. Native MS uniquely operates under non-denaturing conditions, retaining non-covalent interactions into the gas phase. Native MS can provide unique insight into the composition, architecture and function of large biomolecular assemblies and has been used to study, among others, ribosomal particles^3^, membrane protein complexes^4^, virus-like particles^5^ and endogenous viruses^5–7^ (also reviewed in other literature^8–11^).

The mass analysis of macromolecular assemblies is still less efficient than the analysis of smaller molecules (for example, peptides, metabolites and proteins) as these large assemblies suffer from suboptimal transmission through the mass analyser and often generate insufficient signal on a detector. Moreover, most mass analysers exhibit lower resolving power at a higher mass-to-charge ratio (*m*/*z*), which often makes it impossible to resolve sufficient features in the mass spectra of complex or heterogeneous samples. Macromolecular assemblies often represent heterogeneous samples, due to variations in composition, cargo-loading and/or protein post-translational modifications. To better tackle such heterogeneous, high-mass samples, single-particle approaches have emerged in MS, circumventing the need to resolve convoluted ion signals. Two such single-particle approaches are charge-detection MS (CDMS)^12,13^ and nanoelectromechanical systems MS^14^. In CDMS, an independent measure of the charge, *z*, is made, overcoming a major bottleneck in charge state assignments in crowded *m*/*z* spectra. In nanoelectromechanical systems MS, the ions are deposited on small resonators, and a mass for each ion is derived by detecting the resonator frequency shift caused by the deposition of the particle. Both of these approaches have provided impressive results for highly heterogeneous samples like exosomes or viral particles at up to 100 MDa (refs. ^15–17^).

More recently, two groups have in parallel demonstrated CDMS on an Orbitrap-based mass spectrometer by utilizing the linear dependency between the ions’ charge, the induced imaging current of the time domain signal and the resulting peak height in the Fourier-transformed mass spectra^18,19^. CDMS using the Orbitrap analyser offers the possibility to investigate in depth the behaviour of single macromolecular ions within the mass analyser, which is exciting as most of our current knowledge about ion behaviour in the orbital trap is derived from ensemble measurements of smaller and/or denatured particles^20^. Here we demonstrate that such knowledge is beneficial for further progress in native MS and CDMS applications on samples of ultra-high mass.

Of particular impact on the sensitivity and quality of Orbitrap mass spectra is the loss of ion signals during the mass analysis period, whereby ions are lost due to unstable trajectories arising from metastable decay, from space-charge effects or via collisions with background gas molecules. A single collision of a low *m*/*z* (below ~2,000) denatured protein or peptide ion with background gas typically results in the complete loss of the ion within the orbital trap^20–22^, making ultra-high-vacuum (that is, <1 × 10^–9^–1 × 10^–10^ mbar) conditions a necessity. For lower *m*/*z* ions, the energy transfer in such collisions is high enough to cause fragmentation and expel the ions from their coherent motion, causing them to go into unstable orbits^21^. The probability of ions colliding with neutral gas molecules depends on the pressure in the trap, collisional cross-section (CCS) and the travelled distance during the trapping event. The travelled distance largely depends on the trap dimensions, recorded transient time and oscillation frequencies of the analyte ions.

Compared to the standard conditions typically used for applications such as proteomics and metabolomics, macromolecular analytes in native MS acquire substantially fewer charges during the electrospray ionization process. The resulting high-*m*/*z* ions are less efficiently controlled by the ion guides due to their high inertia, making it much more challenging to achieve sufficient transmission. Efficient transmission of high-*m*/*z* ions in native MS can be improved via additional collisional cooling in the front end of the analyser and in the C-trap^23^. However, such approaches may also lead, through leakage, to an undesired increase of pressure in the Orbitrap analyser. In theory, this elevated pressure could be a major bottleneck for maintaining stable ion trajectories over longer transient times, especially considering that macromolecular ions also have higher CCSs. Nonetheless, the first Orbitrap-based single-particle MS studies revealed that surprisingly high stabilities were observed for high-mass ions, even when recording transients of one second^19^.

Here, we investigate the extraordinary stability of these macromolecular ions in the Orbitrap analyser. We find that such ions can easily survive multisecond-long transients, notwithstanding the fact that they experience a multitude of collisions with background gas molecules during that time. The energy deposited by these collisions does not cause fragmentation, as is the case for small and denatured ions, but does lead to gradual solvent loss accompanied in rare instances by charge stripping events. These events are the underlying processes of temporally unstable frequencies, resulting in Fourier transform artefacts (upon processing of the transient by the enhanced Fourier transform (eFT) technique), impairing the performance of Orbitrap-based CDMS.

To characterize the behaviour of each single ion in the Orbitrap analyser, we adapted and built upon a well-known ‘frequency-chasing’ method, previously employed to eliminate frequency drifts and/or improve the measurement of single highly charged, denatured ions (with *m*/*z* < 4,000) using Fourier transform ion cyclotron resonance (FT-ICR) mass analysers^24–26^. We modify and extend this frequency-chasing method to monitor ion behaviour of single macromolecular ions (in the megadalton range), ionized by native MS and analysed by using an Orbitrap at *m*/*z* values well beyond 10,000 Th. Ion motion of these heavy ions in the Orbitrap analyser occurs at energies that are orders of magnitude higher than in high-field FT-ICR; therefore, many of the earlier observations and findings from FT-ICR are not directly applicable here.

Using this modified frequency-chasing method allowed us to extend the transient recording times even further while minimizing Fourier transform artefacts, revealing mechanisms of signal decay that are distinct from those observed in a FT-ICR. Such long transient recording additionally enabled the first ever experimental observation of the radial ion motion in an Orbitrap analyser. Moreover, based on our observations of the behaviour of single ions within the mass analyser, we introduce multiple improved data acquisition strategies for Orbitrap-based CDMS. In combination, these improvements lead to a substantial improvement in effective ion sampling, resulting in better statistics and, by harnessing longer transient data, an almost twofold increase in mass resolution.

Although it is still in its infancy, we foresee that ultrasensitive analysis of macromolecular assemblies by single-particle, Orbitrap-based CDMS will develop into an essential research tool. Research in CDMS to date has focused primarily on virus analysis. Viruses are generally of interest, with nowadays especially those that are used in vaccines and/or gene delivery vectors (for example, adenoviruses and adeno-associated viruses) receiving special attention. Therefore, the improvements in Orbitrap-based CDMS proposed here will benefit applications in virology, biotechnology and gene delivery. As such, analyses can now be performed on instruments that are already widely used within the research community, which will likely open up CDMS-based single-particle analysis to a broader user base.

## Results

### In Fourier transform MS mass resolution and signal-to-noise scale with transient recording times

**Fig. 1:**
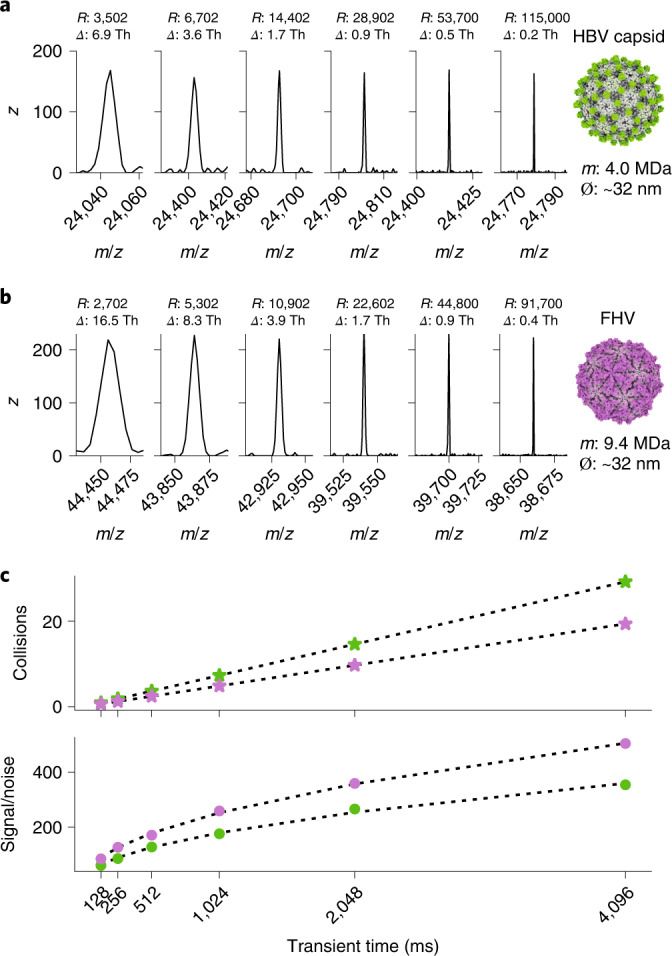
Mass resolution and signal-to-noise in Orbitrap-based CDMS scale with the transient recording time. **a**, Ion signals of individual ions for HBV at increasing transient times of 128, 256, 512, 1,024, 2,048 and 4,096 ms (left to right), with a mass resolution for the 4 MDa particles extending from ~3,000 at 128 ms to above 100,000 at 4,096 ms (1 Th = 1 Da *e*^–1^). *Δ* means the full-width at half-height of the peak. Ø means the diameter of the virus particle. **b**, As in **a**, signals of individual ions of the 9.4 MDa FHV particles, with *R* approaching 100,000 at 4,096 ms. **c**, Average number of collisions the ions experience with background gas (xenon) during the transient time (top), with the green dots showing data for HBV and the purple dots for FHV. Although the number of collisions increases linearly, nearly all high-mass ions seem to survive, as evidenced by the observed resolution and signal-to-noise ratio (bottom) that scale exactly as expected with transient time, reaching a value of ~400 for the 9.4 MDa FHV particles at 4,096 ms. Therefore, for high-mass ions (molecular weight > 1 MDa), even longer transient times would lead to even higher resolution and signal-to-noise levels. Source data

In image current-based detection, as employed in Fourier transform MS, prolonged recording of stably oscillating ions should lead to a higher mass resolution^27^. Whereas in conventional ensemble-based native MS, the effective resolution is a convoluted result of overlapping isotopes, solvent adducts and true resolving power of the mass analyser^27^, single-ion detection enables the precise assessment of the instrument’s performance by analysing individual ion signals, thereby avoiding the detrimental effects of isotope interferences. For truly stable oscillating ions, there should be a linear trend of increased mass resolution with longer transient times. Here we explored whether this is indeed the case. We analysed single ions of the 3–4 MDa hepatitis B virus (HBV) capsids and ~9.4 MDa Flock House virus (FHV) in the Orbitrap analyser at several transient recording times between 128 ms and 4,096 ms under minimized pressure settings (Fig. 1). For all of these particles, we observed the expected linear increase in resolution (*R*) with transient recording time, with *R* increasing from ~3,000 at 128 ms to around 100,000 at 4,096 ms. This level of mass resolution is unprecedented for particles of this size and makes it theoretically possible to resolve mass differences of less than 9 ppm. This corresponds to a change in measured mass of 40 Da and 90 Da for HBV and FHV, respectively, or around 0.001% of the mass of the analyte.

These multisecond-long stable trajectories of individual ions are quite remarkable, as the average number of collisions they experience also scales linearly with the transient length. It can be estimated that these macromolecular particles experience around 30 collisions on average with background gas molecules during a ~4 s transient time (Fig. 1c). For individual ions, the signal-to-noise ratio can be used as a proxy for ion survival as it will be reduced when the ion is lost or absent from later segments of the recorded transient. The plotted signal-to-noise ratio in Fig. 1c follows, for both the HBV and FHV particles, the theoretically predicted square root dependency on the transient duration, confirming that these high-mass ions survive and are stable over the whole transient time, despite undergoing numerous collisions.

### A plateau of stability is reached in the Orbitrap analyser for megadalton particles

**Fig. 2:**
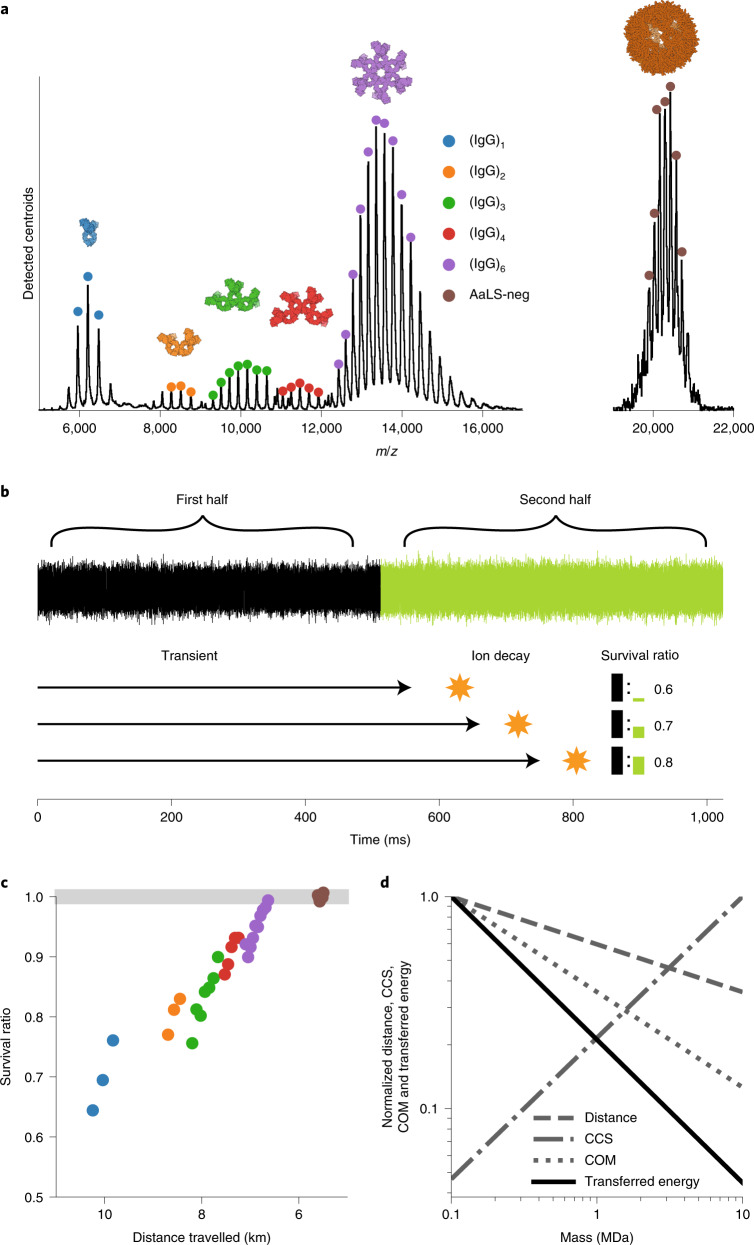
Exquisite high stability for megadalton particles in Orbitrap-based native MS. **a**, Native MS spectrum constructed from binned detected centroids for the IgG1-RGY oligomers (that is IgG, (IgG)_2_, (IgG)_3_, (IgG)_4_, (IgG)_6_) and the nanocontainer AaLS-neg, both recorded at similar settings. Ion signals of different particles are colour-coded as annotated. Dots indicate charge states selected for survivability analysis. **b**, Comparing ion intensity between the first and second half of the transient is used as a proxy for ion survivability. This process is illustrated with ions that decay during different stages of the second half of the transient, resulting in varying ratios for the detected ion signals (black and green bars). **c**, Comparison of ion survival ratio for different charge states of different IgG oligomers and AaLS-neg using a colour code as in **a**. **d**, Favourable scaling of normalized travelled distance, centre-of-mass (COM) and CCS with increasing mass results in sharply reduced energy transfer per surface area, responsible for the plateau of stability reached for megadalton particles. Values on the vertical axis are shown relative to those for a hypothetical particle with a mass of 100 kDa. Source data

To further understand the distinctive stability of megadalton particles compared to smaller protein ions, we analysed single ions of a series of successive IgG1 oligomers (termed IgG1-RGY). The engineered IgG1-RGY variants have the merit of forming in solution oligomers from monomers up to hexamers, yielding ions across a wide mass range within one sample (with molecular weights of ~150, 300, 450, 600 and 900 kDa)^28,29^. Furthermore, we included in our analysis an even higher mass particle, the 3 MDa engineered nanocage AaLS-neg^30,31^. The binned detected centroids for both IgG1-RGY and AaLS-neg are depicted in Fig. 2a. We used a segmented Fourier transform analysis^32–36^ of the recorded transients, dividing them in two halves, to estimate ion survival ratios in a semiquantitative manner (Fig. 2b). The ratios in signal between the first and second segments, here termed ‘survival ratio’, are presented in Fig. 2c, where we converted the respective *m*/*z* positions to distance travelled over the ~1 s transient. This analysis evidently shows that a high percentage of the monomeric IgG1 ions (~30%) do not survive for the entire 1 s transient time, whereas for the higher IgG1 oligomers, the survival ratio increases substantially and continually. We also observe improved survival for lower charge-state ions within distinct oligomers and a general trend of improved survival for larger oligomers at the same travelled distance. The differences between charge states of the same oligomer are driven not only by the increased path length for higher charge states, as the collision probability scales linearly with the travelled distance, but also by the change in transferred centre-of-mass energy, which scales inversely with the *m*/*z* ratio. The differences in survival between oligomer species at similar travelled distances (for example, at 8.5 km travelled distance) is the combined result of an increased CCS and decreased centre-of-mass energy (Supplementary Fig. 1 for further information). The favourable scaling of travelled distance, centre of mass and CCS as a function of mass results in a sharply decreased amount of transferred energy per surface area for high-mass samples, as illustrated in Fig. 2d. This, in combination with the additional degrees of freedom for energy redistribution, is most likely the main driving force for the higher survival rate of the larger particles. Interestingly, these latter particles seem not to show any sign of decay in spite of the multiple collisions they experience over the course of the transient time.

### Gradual solvent loss results in a frequency drift of high-mass ions in the Orbitrap analyser

Above we demonstrated that single megadalton particles can be successfully and stably trapped for durations as long as several seconds, allowing astonishing resolution and precision in mass analysis. Still, while analysing signals of thousands of single ions, we also recorded single-ion signals that deviated from the typical Gaussian peak shape, and for a few of them extensive peak splitting was observed after Fourier transform employing the eFT approach^19^. We hypothesized that this processing artefact is indicative of other gas phase mechanisms especially affecting high-mass ions and could constrain the use of longer transients. Consequently, we decided to dissect the recorded transients into much shorter segments to monitor in time each recorded single-ion perturbation. We term this approach, which builds upon strategies employed earlier by the groups of Smith and Marshall for FT-ICR analysis^24–26^, ‘frequency chasing’, as depicted in Supplementary Fig. 2.

**Fig. 3:**
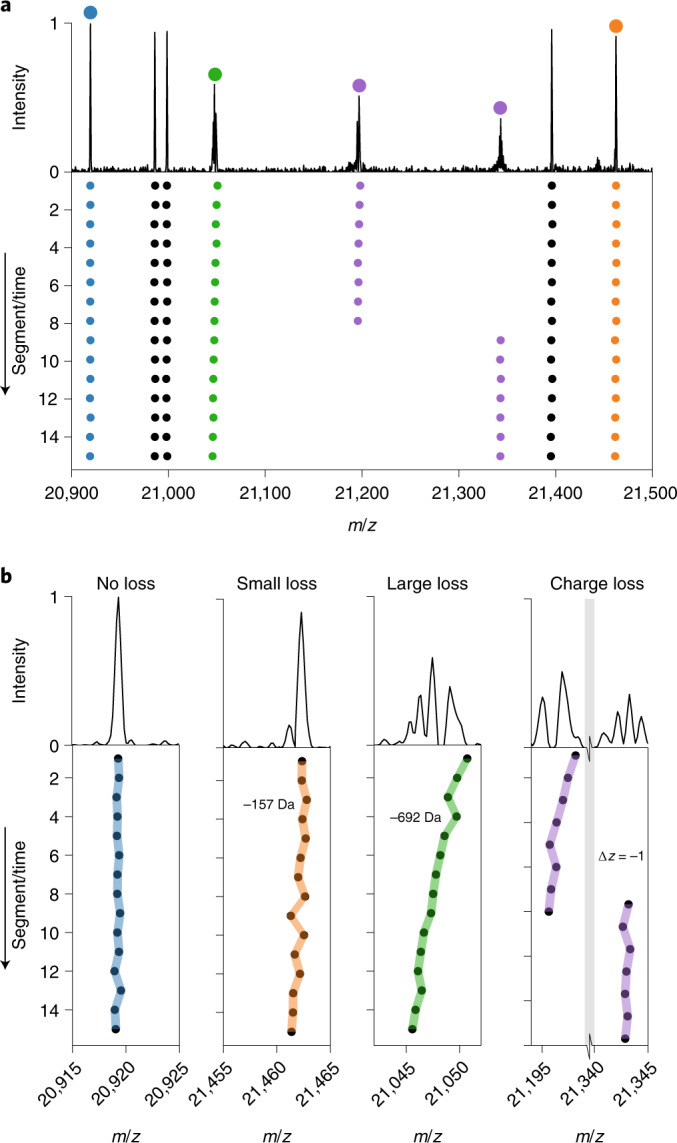
Distinctive behaviour types of single ions within the Orbitrap analyser. **a**, Single-particle mass spectrum with four individual AaLS-neg ions highlighted (colour coded). The 1 s transients were divided into 15 segments and separately analysed. The frequency and thus *m*/*z* of each colour-coded ion over these 15 segments was chased (bottom). **b**, Most single ions displayed no shifts and were stable in frequency and *m*/*z* (95% confidence interval of centroiding is shown as coloured area) over the whole transient (annotated by ‘no loss’). A smaller percentage of the ions displayed gradual frequency shifts, and thus *m*/*z* shifts, due to a gradual increase in desolvation (annotated by ‘small to large loss’). The loss of several neutral molecules (most likely primarily water and ammonium acetate molecules) leads to observed mass shifts of on average 157 Da and 692 Da, respectively. A few ions experienced a quantized jump in frequency and thus *m*/*z* (grey bar indicates broken *x* axis), to higher *m*/*z*, which is attributed to events whereby the ion loses a single charge. Source data

Examples of such segmented Fourier transform analyses, whereby we tracked individual AaLS-neg ions over a ~1 s transient, are shown alongside the recorded eFT mass spectrum in Fig. 3a. The displayed scan is contained in the larger dataset analysed in Supplementary Fig. 3 and highlights the three different categories of peaks we observe consistently in eFT mass spectra of individual ions. These are (1) symmetric peaks without distinct satellite signals, (2) split peaks with satellite signals at lower *m*/*z* and (3) a clear doublet of peaks with a matching peak at a later point in time shifted to a substantially higher *m*/*z* (Fig. 3). Fortuitously, especially at low pressures in the orbital trap, the vast majority of detected single-ion events fall into category (1), whereas type (2) and (3) events are exceedingly rare (Supplementary Fig. 2 also, for quantification). Our frequency-chasing technique enables the evaluation of the stability of single-ion trajectories and the investigation of potential decay mechanisms. Moreover, frequency chasing also allows a quantitative description of centroiding accuracy as a function of transient lengths, which we describe in more detail in the Supplementary Notes and Supplementary Fig. 4.

The symmetric type (1) peaks are completely stable in frequency within the error margin of one Fourier transform bin (denoted here as ‘no loss ions’). Type (2) peaks show a gradual variable shift to lower *m*/*z*, hinting at a gradual consecutive loss of small solvent molecules. These mass losses result in frequency shifts, and when these exceed one Fourier transform bin, eFT processing will produce the observed split peaks with satellite signals at lower *m*/*z*. The type (3) peaks, which usually appear as a doublet, exhibit a sudden jump in frequency (appearing at higher *m*/*z*) corresponding exactly to the shift of −1 charge on an ion of near-identical mass, indicating that they are caused by a single charge-loss event. Notably, these rare events allow a direct charge and mass assessment^37^. The resulting calculated charge state, after the charge-loss event represented in Fig. 3, is 144.07 ± 0.16 (with the ± error propagated from centroiding accuracy), which is a near integer value as expected. From this value of *z* we can directly determine an accurate mass of 3,073,275 ± 29 Da (± error from centroiding accuracy) for this particular single ion observed at a *m*/*z* of 21,343.1 ± 0.2.

**Fig. 4:**
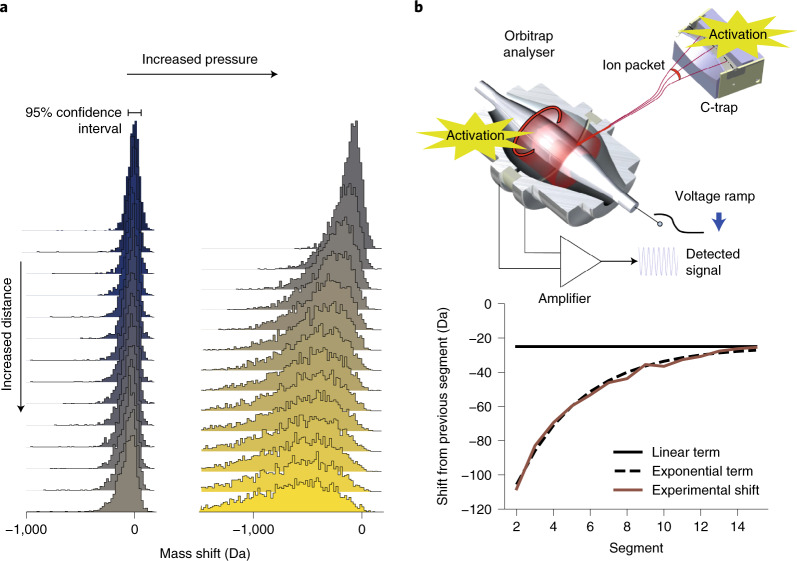
High-mass ion activation and decay within the Orbitrap mass analyser. **a**, Distribution of the observed total neutral loss for individual ions during their transient (top to bottom) at lower and higher pressure read-outs (2.6 × 10^−10^ and 8 × 10^−10^ mbar, left and right, respectively). **b**, Schematic of an Orbitrap mass analyser highlighting regions where ion activation can occur (top). The plot (bottom) depicts the average number of neutral losses relative to the previous segment under the high-pressure conditions. The black solid line indicates the constant loss rate as expected from an activation during Orbitrap detection. By adding a decaying term for the neutral loss, in line with a sudden activation in the C-trap during injection, the experimental loss can be described quite precisely. This analysis clearly reveals the dual nature of the activation process for megadalton particles. Source data

We also monitored the frequency drift behaviour of a large number of individual ions using our frequency-chasing approach (Supplementary Fig. 2) over a wider pressure range to investigate the underlying gas phase processes that govern the observed desolvation and charge-loss events. Figure 4a shows the cumulative loss of individual ions for each segment with respect to the first segment for a large dataset of single-ion recordings. This demonstrates a continual decrease in solvent loss over the duration of the transient, especially in the later segments. We modelled the observed trend of frequency shifts as co-occurring neutral loss processes. The first one, denoted ‘linear’ solvent loss, occurs with a constant rate of collisions per travelled distance in the Orbitrap analyser. These collisions are the underlying cause for the ion decay of smaller and denatured proteins as earlier described by Brodbelt et al.^21^. The second effect presents itself as an ‘exponential’ solvent loss over the transient segments, in line with a suddenly activated system, which attenuates its energy gradually by solvent loss. This activation is most likely happening when the ions are injected from the C-trap into the Orbitrap mass analyser (Fig. 4b). Although the travelled distance is short in comparison with the several kilometres travelled during Orbitrap transient acquisition, the pressure in that region is more than a million times higher (~10^−3^ mbar) relative to orbital trapping conditions, and ions are accelerated by approximately 1 kV. We successfully simulated the solvent loss for all pressure settings using a combination of a linear and exponential decay function (with the square of the correlation coeffcient *r*^2^ between 0.998 and 0.9998). The value used for the ‘linear’ and ‘exponential’ solvent loss terms scale, as anticipated, linearly with the ultra-high-vacuum pressure read-outs in the mass spectrometer, which in turn scales linearly with the high-vacuum pressure gauge read-out close to the C-trap (Supplementary Fig. 3).

### Optimizing Orbitrap-based CDMS of megadalton particles

**Fig. 5:**
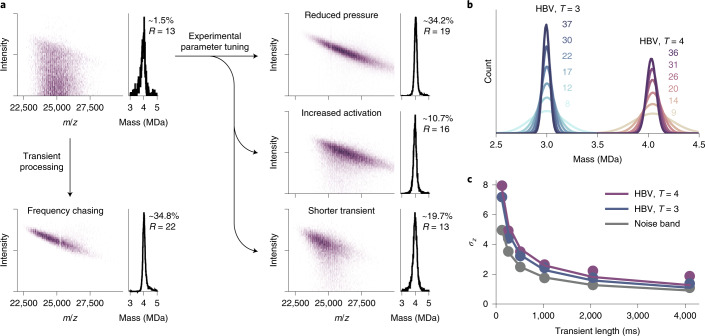
Optimizing sensitivity and resolution in Orbitrap-based single-particle CDMS. **a**, Overview of experimental and data processing approaches that can be exploited to reduce the number of split ion signals, improving CDMS performance, shown for the 4 MDa heavy HBV *T* = 4 capsid. As the arrows indicate, the number of split peak occurrences can be reduced by decreasing the gas pressure (reducing the collision probability), improving the initial desolvation (lower solvent loss probability) and/or diminishing the transient time. Split peaks can additionally be diminished through frequency chasing, applying drift corrections. This may result in a ~23-fold increase in effective ion sampling overall. Each of these approaches results in a much better utilization of the acquired data and yield mass histograms (on the right with percentages of utilized recorded ion signals) with comparable resolutions. **b**, Mass histograms of HBV particles, with *T* = 3 and *T* = 4 (~2,000 ions each), demonstrating the improving mass resolution by combining longer transient times and frequency drift correction (resolution indicated in matching colour and corresponds to 128, 256, 512, 1,024, 2,048 and 4,096 ms total transient times from bottom to top). **c**, Charge resolution in single-particle CDMS as a function of the transient time for HBV, with *T* = 3 and *T* = 4, as well as of the noise band of the corresponding *m*/*z* region. An exponential function (*σ*_*z*_(*t*) = *A* × *t*^*B*^, where *t* is transient time, and *A* and *B* are constants) was fitted to the data points and follows a square root (with *B* of −0.52, −53 and −0.49; and *r*^2^ of 0.98, 0.99 and 1.00; for HBV with *T* = 4 and *T* = 3 and for the noise band), indicating that the electronic noise of preamplifier transistors is the main contributor to the observed impaired charge-resolving power. Source data

The core principle of Orbitrap-based CDMS is that single-ion intensities scale linearly with the ion’s charge. Consequently, peak splitting caused by pressure-dependent neutral losses represents a major bottleneck for the analysis of megadalton particles, especially considering that such analytes require a certain amount of gas present to facilitate effective transmission and desolvation. However, with the new insights into high-mass ion behaviour, we explored several experimental and ad hoc processing approaches to address temporal frequency instability as presented schematically in Fig. 5a. In this figure for ions of the HBV *T* = 4 capsid (where *T* stands for triangulation number), the CDMS data on the top left were recorded at a relatively higher pressure, a condition under which the eFT peak splitting occurs frequently, which substantially limits the sampling rate of ions when appropriate filtering of the split peaks is applied (~1.5% signal utilization). This effect can be minimized by choosing suitable experimental conditions preventing either neutral loss or peak splitting. Since collisions with neutral gas molecules seem to be the main driver for neutral losses, optimizing the pressure settings can improve the relative amount of ion sampling by a factor of ~23. Also, better desolvation of particles by increased collisional activation reduces neutral loss and peak splitting and thus increased ion sampling by a factor of approximately seven. This is most likely caused by the larger amount of energy these remaining solvent molecules need to evaporate. Additionally, a reduced transient duration can reduce peak splitting by the combined effect that less time is spent in orbit, where the collisions and neutral losses take place, but also by the larger Fourier transform bin widths, which accommodate more frequency drift before splitting. This improves ion sampling by a factor of ~13.

Lastly, if experimental conditions do not allow further optimization of the above-mentioned parameters, frequency drifts can be corrected using the output of our ‘frequency-chasing’ method. By taking the individual segments, as shown in Fig. 2, the drift in *m*/*z* position is so small relative to the Fourier transform bin width that the top peak intensity stays unaffected. By subsequently averaging the intensity values of each of the segments, a similar resolution can be achieved as that for stable frequencies over the whole transient time. This approach allows the recovery of a large portion of the split peaks shown in Fig. 5, resulting in an ~23-fold increased utilization of the recorded ion signals.

With the use of our above-described frequency-chasing methods, it is possible to accommodate the desire to record even longer transients while mitigating the effects of frequency drifts to possibly improve performance. This would not be possible with eFT-based filtering since the underlying source of such drifts (that is, collisions with background gas) increases, while the tolerance for peak splitting (width of the Fourier transform bin) decreases with the transient time. In Fig. 5b, we show the resulting mass histograms of HBV. HBV capsids can coexist in two formations, with *T* = 4 and *T* = 3 symmetry, containing 240 and 180 capsid proteins and forming particles of about 4 and 3 MDa, respectively^38^. For the data shown in Fig. 5b, we utilized increasing portions of a 4,096 ms transient while applying frequency chasing on approximately 2,000 individual particles for HBV. The mass distribution of these ions displays a successive increase in resolution for increasing utilized portions of the transient, while omitting the detrimental results from peak splitting. Since the mass resolution is mainly limited by the resolution in the charge dimension, a similar trend can be seen for the spread in the intensity domain in Fig. 5c. The similar scaling of the intensity spread, as well as the noise band, identifies electronic noise of preamplifier transistors, amplified by the relatively high capacitance of the Orbitrap electrodes, as the main contributor of non-perfect charge assignments. This noise, as well as the spread in the intensity dimension, improves, closely following the expected square root scaling with the transient lengths until 2,048 ms. The deviation of the noise band is rather small and can be attributed to electronic ringing or other background processes and the drift of voltages for such long transients. The larger deviations (from the theoretical square root behaviour) for the actual single-ion intensities compared to the noise band suggest additional artefacts associated with ion behaviour rather than pure instrument performance. Besides sample heterogeneity in the HBV capsid assemblies^39^, these artefacts are most likely caused by the wide range of kinetic energies accepted by the Orbitrap as well as eccentric, non-circular orbits, both impacting the radial distance and thus the induced image current^40^.

**Fig. 6:**
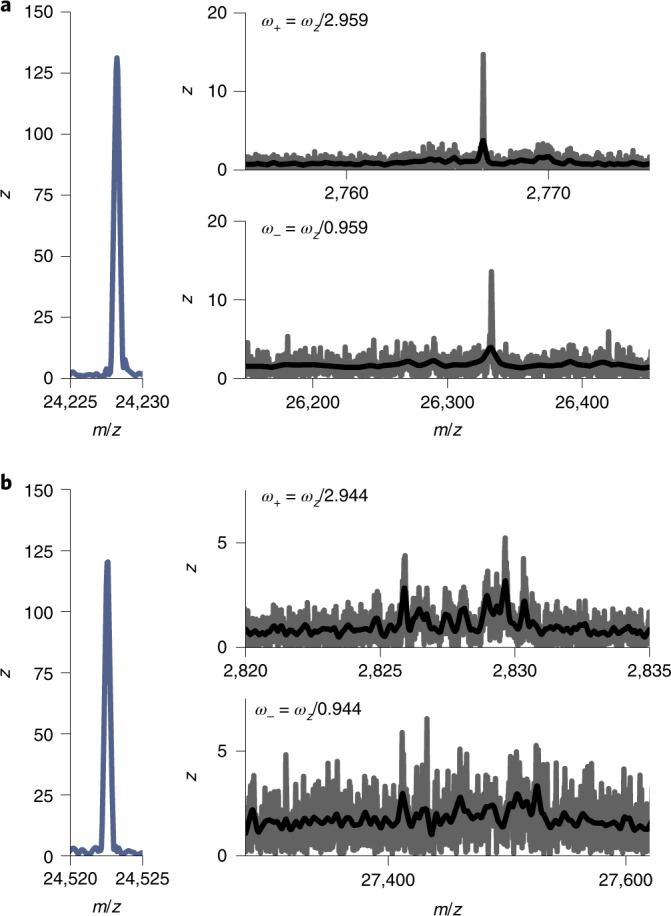
Detecting radial ion motion in an Orbitrap analyser. **a**,**b**, The detected peaks for two individual instances of HBV *T* = 3 single ions. On the left, the peak at the standard axial frequency (*ω*_*z*_) is displayed (shown in the *m*/*z* domain). On the right, signals originating from the radial frequency modulations, *ω*_+_ and *ω*_–_ (at higher and lower frequency with respect to *ω*_*z*_, respectively) are displayed in the lower and higher *m*/*z* region (top and bottom). The raw data is shown in grey, the smoothed signal in black. The ion in **a** illustrates a highly stable radial frequency, whereas the single ion in **b** represents a non-stable radial frequency, for example caused by non-circular orbits. Note that in **b** the signals originating from the modulation by *ω*_+_ and *ω*_–_ are, as expected, mirror images of each other in the frequency domain. Source data

As an additional benefit, the recorded longer transients of highly charged particles with a high signal-to-noise ratio enable us to observe and detect the radial frequencies through their modulation of the axial ion frequency, experimentally validating their earlier theoretical description^41^. To illustrate this, in Fig. 6 we depict two instances of CDMS data on single ions where we highlight the corresponding regions at higher and lower *m*/*z*, displaying a stable as well as an unstable radial frequency. Notably, the resulting shapes of the frequency modulations mirror themselves in the low- and high-*m*/*z* regions, verifying that the two modulations are caused by the same radial oscillation (Supplementary Fig. 5 for a similar observation of radial frequency modulations for single ions of the bacterial chaperone GroEL and the FHV, and the Supplementary Notes for further theoretical and experimental description of these radial oscillations).

## Discussion

The foregoing detailed analysis of the behaviour of individual ions of megadalton particles within the Orbitrap analyser, including the analysis of different-length Fourier transform segments, provides valuable insights allowing us to stipulate a perspective on the future of Orbitrap-based CDMS. As CDMS has unique applications in various important research areas, including structural biology, nanomaterials, fibres, vesicles, vaccines and human gene therapy (for example, adenovirus and adeno-associated virus gene delivery vectors), we address the need for future optimizations towards higher resolution and experimental feasibility, for which we here laid out a fundamental framework.

The observed plateau of stability for megadalton particles was unexpected and seemingly in contrast with the ion behaviour observed for small and denatured ions, where one collision causes ion loss through fragmentation and ion decay. Although such collisions occur frequently when analysing megadalton particles, the deposited energy is attenuated by gradual solvent loss rather than fragmentation, allowing ions to be retained in their orbits for several seconds and possibly even for minutes, if the electronics and hardware would allow us to perform such measurements. The detailed understanding of these activation processes improved CDMS experimental designs, boosting ion sampling by a factor of more than 20 (Fig. 5). Furthermore, with the frequency-chasing method presented here, it is possible to extend the transient time theoretically indefinitely while minimizing the effect of frequency drifts. This is imperative as we expect the Orbitrap mass analyser, extrapolating from the noise band, to have the capability to resolve individual charges at 16 s (standard deviation *σ* = 0.5) and produce an almost perfect charge assignment at 32 s (*σ* = 0.3)^42,43^. At such high charge resolutions, it is very likely that the effect of non-circular orbits and variable ion radii on the induced image current will become apparent. With the radial frequency modulations described here, we shed light not only on the ion motion in the otherwise hidden *x*–*z* plane of the Orbitrap mass analyser, but also on the possibility to correct for its effect on single-ion intensities.

Whereas we are currently technically limited to a maximum 4 s transient time on the Q Exactive UHMR platform, developments to overcome this limit will offer the exciting prospect of improved resolution and signal-to-noise ratio, likely beyond the crucial tipping point of determining the charge state of individual ions within the tolerance of a single elementary charge at a commercially available mass spectrometer.

## Methods

### Sample preparation for native MS

The purified megadalton particles analysed were obtained from various collaborators. FHV was provided by E. Jaworski from the A. Routh lab (University of Texas Medical Branch); the AaLS-neg nanocontainer sample was provided by the group of D. Hilvert (ETH Zurich); and IgG1-RGY samples were provided by the team of J. Schuurman (Genmab). HBV dimers were provided by N. R. Watts (National Institutes of Health). FHV, AaLS-neg and IgG1-RGY were buffer exchanged to aqueous ammonium acetate (150 mM, pH 7.5) with several concentration/dilution rounds using Vivaspin Centrifugal concentrators (9,000*g*, 4 °C). HBV capsids were formed by diluting HBV dimers (Cp140) directly into aqueous ammonium acetate (150 mM, pH 7.5; capsids form within minutes). An aliquot of 1–2 μl was loaded into gold-coated borosilicate capillaries (prepared in-house) for nano-electrospray ionization. Samples were analysed on a standard Q Exactive ultra-high mass range instrument (Thermo Fisher Scientific)^7,44^.

### Instrument setting for single-particle native MS

The instrument settings required for the analysis of large assemblies such as ribosomes and FHV have already been described in detail^7,44^. In short, the ion transfer target *m*/*z* and detector optimization were set to ‘high *m*/*z*’, where radio frequency amplitudes for the injection flatapole, bent flatapole, and transfer multipole and HCD-cell were set to 700, 600 and 600 V, respectively, and detector optimization was set to ‘low *m*/*z*’. In-source trapping was enabled with desolvation voltages ranging between −50 and −200 V. The ion transfer optics (injection flatapole, inter-flatapole lens, bent flatapole and transfer multipole) were set to 10, 10, 4 and 4 V, respectively, and values of 7, 7, 7 and 7 V were used for the analysis of the IgG-RGY samples. We used either xenon^7,45^ or nitrogen as the neutral gas in the collision cell, and complexes were desolvated via activation in the higher-energy collisional dissociation cell. Ion transmission was attenuated by diluting and shortening the injection time until individual ions were observed. Data were acquired in a range between 15 minutes to 2 hours, which resulted, depending on the set transient time, in datasets of 500 to 2,000 scans or transients, with on average tens of individual ions in each scan. Transients were saved by setting the corresponding tab to ‘save with RAW file’ in the ‘service’ section of the Tune interface.

### Transient processing

The transients were four times zero-padded (as is customary^46^ in Fourier transform MS signal processing) prior to calculating their fast Fourier transform. The absolute values of the fast Fourier transform (that is, magnitude mode) spectra were converted to the *m*/*z* domain using the instrument calibration parameters. The reported centroids of the observed peaks were calculated via a three-point least squares parabola fit.

To monitor the temporal behaviour of the observed masses^32–35^ (or more accurately, the oscillation frequencies of the ionic species in the Orbitrap analyser) during the transient acquisition, the time domain signals were segmented into a set of overlapping windows^36^. A mass spectrum for each sub-transient was computed as described above, and the masses of the ions of interest were ‘chased’ in time. Further details are also in the Supplementary Information section.

### Ion tracing

To investigate time-resolved ion behaviour, it is necessary to assign centroids appearing in the different segments to the ion they are originating from. Since the resolving power is much lower for individual segments converted via magnitude Fourier transform as compared to the eFT of the whole transient, only ions that were not adjacent to other centroids within a certain distance were traced. This distance is defined by the achievable resolving power at the given segment length and *m*/*z* position. This step also circumvents the possibility of misassigning centroids from two crossing ion species. After removing all closely spaced peaks, each centroid in the first segments was matched with the closest centroid (in the *m*/*z* dimension) of the following segments. The matched centroids are then filtered with regard to their standard deviation in the intensity and *m*/*z* dimensions and subjected to further analysis.

### Frequency chasing

Ion frequency drift analysis, or frequency chasing, was performed on traced and filtered segmented centroid data. In order to convert frequency drifts into neutral losses, all ions were binned in *m*/*z*, and a conventional charge state assignment was performed. Next, each centroid was assigned to a charge based on its *m*/*z* position, and frequency drifts could be converted to neutral losses in daltons. Cumulative neutral losses and losses per segment were calculated with respect to the calculated ion mass in the first or the previous segments, respectively.

### Ion path length, CCS and neutral collision event calculations

Ion path lengths for individual ions were calculated as the product of transient length, frequency and average travelled distance per oscillation along the *z* axis. The transient length is determined by the set instrument resolution, and the frequency was approximated for a given ion following the equation *f* = 0.26055 × (*m*/*z* × 10^−3^)^−0.5^ (MHz). The average travelled distance per oscillation along the central electrode (93.7425 mm) was averaged over ions with different radial distances caused by the wide range of accepted ion energies for ions of the same *m*/*z*.

Collisional cross-sections were calculated using the formula CCS = π × (*r*_i_ + *r*_n_)^2^ ≈ π*r*_i_^2^, with *r*_i_ and *r*_n_ being the radii of the corresponding ion and neutral gas, respectively. The *r*_i_ values were extracted by manually measuring the diameter of the corresponding Protein Data Bank structure (HBV, 1QGT; FHV, 4FTB). The average number of gas molecules in the Orbitrap analyser was calculated from the ultra-high-vacuum read-out of the cold cathode gauge assuming the ideal gas law. We used temperature 25 °C as measured by the temperature probe on the Orbitrap block. The real gas pressure inside the trap is likely about twofold higher than that measured by the cold cathode gauge according to a reference^21^. Additionally, since the installed cold cathode gauges are calibrated based on nitrogen, we applied the appropriate correction factor if used with xenon. The mean free path of a given molecule was calculated by the product of its CCS and the concentration of gas molecules. The average number of collision events for a given ion is the quotient of travelled distance and mean free path.

### Frequency drift corrections

Frequency drifts of ions, resulting in split eFT peaks, were recovered for CDMS by utilizing segmented Fourier transform analysis of the transient. Centroids from individual segments were grouped to ions as described above. These ions were then filtered for instances with a standard deviation above 4 and 0.2 for the *m*/*z* and intensity dimension, respectively. For ions passing these criteria, intensities and *m*/*z* values were averaged over the individual segments. We found that the magnitude of Fourier transform single-ion intensities can be converted under these acquisition conditions into charges by multiplying by a factor of 175.

## Online content

Any methods, additional references, Nature Research reporting summaries, source data, extended data, supplementary information, acknowledgements, peer review information; details of author contributions and competing interests; and statements of data and code availability are available at 10.1038/s41557-022-00897-1.

## Supplementary information

Supplementary Information Supplementary Figs. 1–5, notes and references.

Supplementary Data 1 Description of the code used with an example.

## Data Availability

Data supporting the findings of this study are available within the paper and its Supplementary Information. Source data are provided with this paper. Alternatively, the data are available from the corresponding author upon request. An example dataset is provided alongside the code example as Supplementary Data 1.

## Source data

Source Data Fig. 1 Statistical source data.
Source Data Fig. 2 Statistical source data.
Source Data Fig. 3 Statistical source data.
Source Data Fig. 4 Statistical source data.
Source Data Fig. 5 Statistical source data.
Source Data Fig. 6 Statistical source data.

## Code availability

An example Python script for the frequency drift correction approach presented here is available as Supplementary Data 1.
