# Supplementary material for: Frequency chasing of individual megadalton ions in an Orbitrap analyser improves precision of analysis in single-molecule mass spectrometry
Source: Nat Chem. 2022 Mar 10;14(5):515–22. doi: 10.1038/s41557-022-00897-1 (PMC9068510; doi:10.1038/s41557-022-00897-1)
Supplement: Supplementary file 1 — Supplementary Figs. 1–5, notes and references. [file 41557_2022_897_MOESM1_ESM.pdf]

nature portfolio

https://doi.org/10.1038/s41557-022-00897-1

Supplementary information

Frequency chasing of individual megadalton ions in an Orbitrap analyser improves precision of analysis in single-molecule mass spectrometry

In the format provided by the authors and unedited

Supplementary Information to:

Frequency chasing of individual megadalton ions in an Orbitrap analyzer improves precision of analysis in single molecule mass spectrometry

Authors:

Tobias P. Wörner[1,2], Konstantin Aizikov[3], Joost Snijder[1,2], Kyle L. Fort[3], Alexander A. Makarov[1,3] & Albert J.R. Heck[1,2,*]

Affiliations:

[1] Biomolecular Mass Spectrometry and Proteomics, Bijvoet Center for Biomolecular Research and Utrecht Institute for Pharmaceutical Sciences, University of Utrecht, Padualaan 8, 3584 CH Utrecht, The Netherlands;

[2] Netherlands Proteomics Center, Padualaan 8, 3584 CH Utrecht, The Netherlands;

[3] Thermo Fisher Scientific (Bremen), Bremen, Germany;

* Corresponding author: A.J.R.Heck@uu.nl

Supplementary Notes

Accuracy of centroid determination

Detailed investigation of the behavior of megadalton single particle ions create a unique opportunity to look into their spectral idiosyncrasies from the signal processing perspective. In particular, the data exposes limits of peak centroiding accuracy in FTMS for shorter transient as well as under the elevated noise conditions[1,2]. In the current investigation all the centroids were determined using a parabola fit to the peaks in the magnitude spectra of the four times zero-padded, cosine-apodized time domain signals. This procedure was used for analyzing both, the entire transients as well as their constituent segments. The signal-to-noise (S/N) was defined as the ratio of an observed peak apex to the σ of the spectral noise. For numerical experiments on the temporal stability of the ion frequency, a purely synthetic non-decaying harmonic signal with no modulations of any kind was added, at *m/z* 27130, to a 1-second-long experimental transient of a single ion of a FHV particle at *m/z* 42980 (see Supplemental Figure 4a). These two peaks, experimental and synthetic, were subsequently analyzed for stability over time.

In a first set of the computational experiments a rather short temporal window was taken $\boldsymbol{t_{win}}$= *64 ms*, which guaranteed baseline resolution for the interrogated peaks. The resulting smooth spectrograms (see Supplemental Figure 4a insets) were achieved via stepping with a small (relative to $\boldsymbol{t_{win}}$) overlap of $\boldsymbol{t_{step}}$= *4ms*. The observed frequency modulations for both the experimental and the synthetic peaks appear to be systematic but uncorrelated. In case of the latter it is truly surprising as the artificial signal should not carry any temporal instabilities. If, however, the observed centroids are considered without the temporal information but rather as data points for histograms reporting the deviations to their expected values (see Supplemental Figure 4a insets), they appear as near-bell shaped distributions with full width at half maximum (FWHM) for the synthetic peak of ~49ppm, and that for the experimental peak of ~72ppm in terms of their *m/z*. Although the widths of the histograms differ in terms of ppm of their respective *m/z*'s, the peaks come from the different regions of the underlying frequency spectrum with intrinsically different granularities (i.e. for the 27130 Th (Da/e) peak the FT bin is 4.35 Th

(~160.44 ppm), and that for the 42980 Th is 8.68 Th (~201.93 ppm)), and the reported spread is substantially smaller than the uncertainty imposed by the FFT grid. This all suggests that the observed mass errors are the result of distortion of spectra by noise.

Failing to observe any significant modulations in the experimental peak beyond the centroiding uncertainty (based on the behavior of the synthetic component), it is worthwhile to look into the dependency of the frequency deviation spreads on the sub-transient duration $\boldsymbol{t_{win}}$. In the next experiment the same frequency chasing experiments were performed on the same two peaks for different $\boldsymbol{t_{win}}$. The findings are summarized in Supplemental Figure 4b. Unlike the peak intensity uncertainty, which depends directly on the noise levels, the $\boldsymbol{\sigma}$ of centroiding follows the negative power trend:

$$\sigma = a{t_{win}}^{-p}. \quad (1)$$

We expect both **a** and **p** to be *m/z* dependent; the investigation of this dependency is however beyond the scope of this manuscript. The observed values are $a_{42980} = 0.025$, $a_{27130} = 0.009$, and those for the exponents are $p_{42980} = 1.45$ and $p_{27130} = 1.54$. The differences scale well with the respective frequency spectrum region granularities. The overall centroiding uncertainty is well explained by noise. The apparent regularity in the observed deviations (see Supplemental Figure 4a insets) is an artifact of the finesse of sampling $\boldsymbol{t_{step}}$. Only if the $\boldsymbol{t_{win}}$ is extrapolated to 2 seconds (and longer) using the observed relationships ($\boldsymbol{\sigma_{27130Th}}$ = 0.0028 Th & $\boldsymbol{\sigma_{42980Th}}$ = 0.0087 Th) the ratio between the exponential and synthetic σ's become larger than one would expect based solely on the FT grid spacing suggesting that there are underlying physical phenomena (*e.g.* ion motion, space and image charge, *etc*.) driving the discrepancy.

The fidelity of this methodology (as that of FTMS) directly depends on the accuracy with which one can extract the frequency information (or equivalently *m/z*), where precision of peak centroiding is probably the most important step. The two biggest sources of ambiguity identified so far are the noise and the non-homogenous granularity of the Orbitrap mass spectrum. As the spacing between the data points (aka bins) in FTMS spectra tend to increase with mass, this adds to the uncertainty in accuracy of centroiding and, of course, the ambiguity brought about by noise. We focused on the effects of these two factors on the σ of the centroids. A set of 2D shotgun type experiments was conducted

by injecting purely synthetic peaks into the experimental noisy transient across the mass range commonly used for viral particle analysis (*i.d.* 20-50kTh) stepping every 50 Th (to ensure thorough coverage); the S/N (defined as the ratio of a peak intensity to noise *σ*) used was ranging 6-60 with 0.2 AU step. The results are shown in Supplemental Figure 4c. As expected for sparse and baseline resolved spectra, the centroiding uncertainty increases with the noise levels, and, as was observed previously, with *m/z*. However the increase in *σ* was correlated with the mass grid spacing (a single FT bin at 20000 *m/z* = ~0.67 Th and at 50000 *m/z* = ~2.66 Th), *i.e.* ${\boldsymbol{\sigma}_{@50K}}/{\boldsymbol{\sigma}_{@20K}} \cong 2.66/0.67$.

Another parameter which affects the centroiding directly is the resolution. There are a number of factors known to affect the peak shape in FTMS (*e.g.* frequency instabilities, ion packet dephasing, and ion loss, *etc*.),[3–7] yet the easiest way to manipulate the peak width is by altering the time domain signal duration. To a degree this was already done in the frequency stability analysis using different transient lengths (see Supplemental Figure 4b); here, the findings are augmented with more focused experiments. The choice was also for a 2D shotgun type experiments where, similarly, the mass was varied from 27000 Th to 27200 Th in 1 Th increments; the transient time, on the other hand, varied by power increments: 2^{-5}, 2^{-4}, 2^{-3}, 2^{-2}, and 2^{-1} of 1.024 Sec. The side-by-side comparison of the results of these *in silico* experiments with the frequency chasing experiments (for the synthetic 27130Th peak) is shown in Supplemental Figure 4d. The shotgun experiments also show the exponential dependency $\sigma_{shotgun} = 0.008 t_{win}{}^{-1.50}$. The trends are very close and made extrapolation of the "temporal evolution" experimental trend up to 0.5 sec long transients possible (see Supplemental Figure 4d). The observed discrepancy between the two is most likely caused by fewer independent data points in the chasing experiments, since it is imperative for that type of experiments to have $t_{win} \ll t_{transient}$.

Experimental observations in the Orbitrap analyzer of theoretically predicted radial frequency modulations

The theoretical ion motion in the Orbitrap analyzer has been described earlier in quite some detail and combines, for a stable trajectory, rotation around as well as oscillation

along the central electrode[8]. This results in a spiraling motion of the ion when injected into the orbital trap and separate terms for the frequencies of oscillations along the z (ω_z) and the polar coordinates r (ω_r) and φ (ω_φ) can be formulated. The image current is detected on the split outer electrodes and the resulting signal, after a differential amplifier, is subjected to Fourier transformation. Attributed to this geometry, the final mass spectrum is calculated based on the frequencies along z. The use of oscillation frequency along z is caused by its independence on of the ion energy. This is crucial as, based on their spatial distribution in the C-trap, ions get injected with a variable amount of energy into the Orbitrap analyzer and thus occupy slightly different radii. For an ion cloud of a given *m/z*, this also means that ions will lose radial coherence rather quickly and the ions will occupy a rotating ring with a certain thickness instead of a defined point in time and space. For ensemble measurement this offers a distinct advantage since the Orbitrap analyzer can accommodate a wider range of ion energies, making it less susceptible to detrimental effects of space charging. However, for individual ions, the variable injection energy and resulting radial orbit may be problematic as the induced image current is inversely proportional to the distance of the ions to the outer electrode cups.

As mentioned in the main text, we report here the first experimental measurements of the radial frequency of ions in the Orbitrap mass analyzer. These radial harmonics are indirectly detected, as they are components of and modulate the total signal:

$$S(t) = \cos(\omega_z t) * [1 + C * \cos(\omega_r t)] \quad (4)$$

and

$$S(t) = \cos(\omega_z t) + C/2 * [\cos(\{\omega_r + \omega_z\}t) + \cos(\{\omega_r - \omega_z\}t)] \quad (5)$$

Therefore, we should find for each ion primary signal two radial frequency modulations at both higher ($\omega_- = \omega_r - \omega_z$) and lower ($\omega_+ = \omega_r + \omega_z$) *m/z* (as shown in Figure 6 and Supplementary Figure 5). We observed radial frequency modulations for all theoretical expected cases like[9]: 1) highly dephased radial frequencies caused by non-circular orbits for ions with poorly matched ion energies. 2) Stable and defined radial frequencies for ions with defined orbit and well matching injection energy. 3) Varying radial frequencies

for the same frequency along z. 4) Slightly reduced radial frequency for lower *m/z* species caused by an earlier arrival in the Orbitrap analyzer and thus more extended orbit.

References

1. Posener, D. W. Precision in measuring resonance spectra. *J. Magn. Reson.* **14**, 121–128 (1974).

2. Chen, L., Cottrell, C. E. & Marshall, A. G. Effect of signal-to-noise ratio and number of data points upon precision in measurement of peak amplitude, position and width in fourier transform spectrometry. *Chemom. Intell. Lab. Syst.* **1**, 51–58 (1986).

3. Makarov, A., Denisov, E. & Lange, O. Performance evaluation of a high-field orbitrap mass analyzer. *J. Am. Soc. Mass Spectrom.* **20**, 1391–1396 (2009).

4. Gorshkov, M. V., Fornelli, L. & Tsybin, Y. O. Observation of ion coalescence in Orbitrap Fourier transform mass spectrometry. *Rapid Commun. Mass Spectrom.* **26**, 1711–1717 (2012).

5. Kozhinov, A. N., Zhurov, K. O. & Tsybin, Y. O. Iterative Method for Mass Spectra Recalibration via Empirical Estimation of the Mass Calibration Function for Fourier Transform Mass Spectrometry-Based Petroleomics. *Anal. Chem.* **85**, 6437–6445 (2013).

6. Ledford, E. B., Rempel, D. L. & Gross, M. L. Space charge effects in Fourier transform mass spectrometry. Mass calibration. *Anal. Chem.* **56**, 2744–8 (1984).

7. Jeffries, J. B., Barlow, S. E. & Dunn, G. H. Theory of space-charge shift of ion cyclotron resonance frequencies. *Int. J. Mass Spectrom. Ion Process.* **54**, 169–187 (1983).

8. Makarov, A. Electrostatic Axially Harmonic Orbital Trapping: A High-Performance Technique of Mass Analysis. *Anal. Chem.* **72**, 1156–1162 (2000).

9. Hu, Q. *et al.* The Orbitrap: a new mass spectrometer. *J. Mass Spectrom.* **40**, 430–443 (2005).

Supplementary Figures

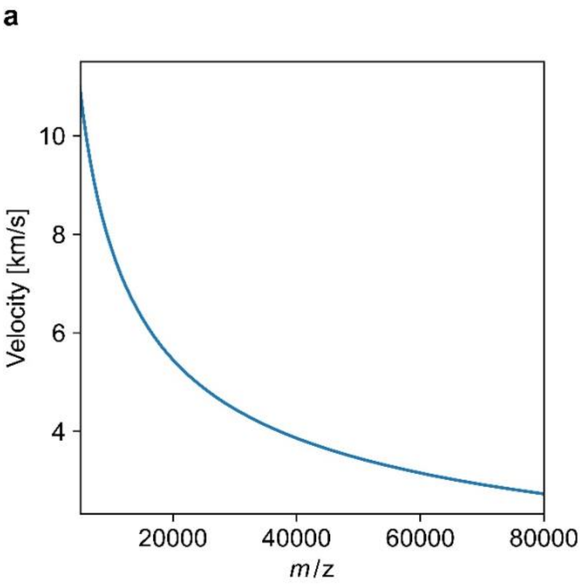

Supplemental Figure 1: Velocity (**a**) and kinetic energy (**b**) of ions trapped in the Orbitrap mass analyzer assuming normal ESI charging of globular proteins.

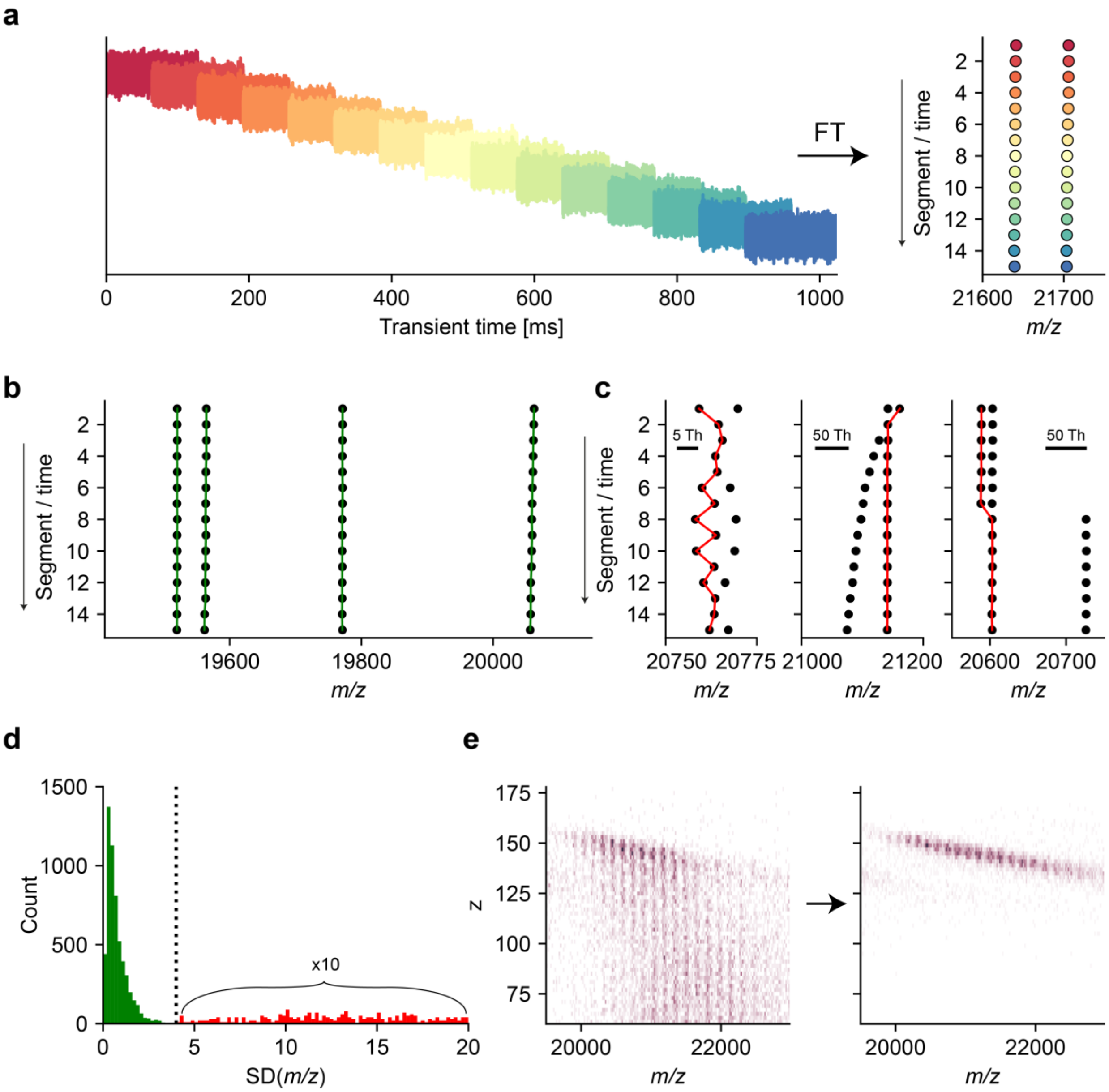

Supplemental Figure 2: a, Illustration of used overlapping transient segments in the frequency chasing method and the resulting centroids per segment after FFT. **b,c,** Examples of ion tracing for a stable case (**b**) where all ions can be traced easily as well as a more complicated case where some ions cannot be traced due to (left to right in **c**) signals which cannot be resolved properly at the given segment lengths as well as crossing ion signals and rare charge loss events (at around 0.5% of all ion events for the given pressure setting). **d,** Histogram of the standard deviation (SD) of the average *m/z* position for traced ions where the green distribution reflects the natural pressure dependent frequency drift as well as much higher values (in red) which reflect rare occasions of falsely traced ions. The spread in SD of the successfully traced ions can also give an impression of the peak splitting prevalence as SD of truly stable ions should not exceed theoretical SD based on centroiding (>0.2Th for 128ms at 20k *m/z*), thus the majority of the peaks for the given pressure setting should exhibit some signs of peak splitting. We applied a filtering step allowing only ions under a certain threshold to be used for further analysis. **e** Illustration of the cumulative effect of frequency chasing on single particle CDMS experiments.

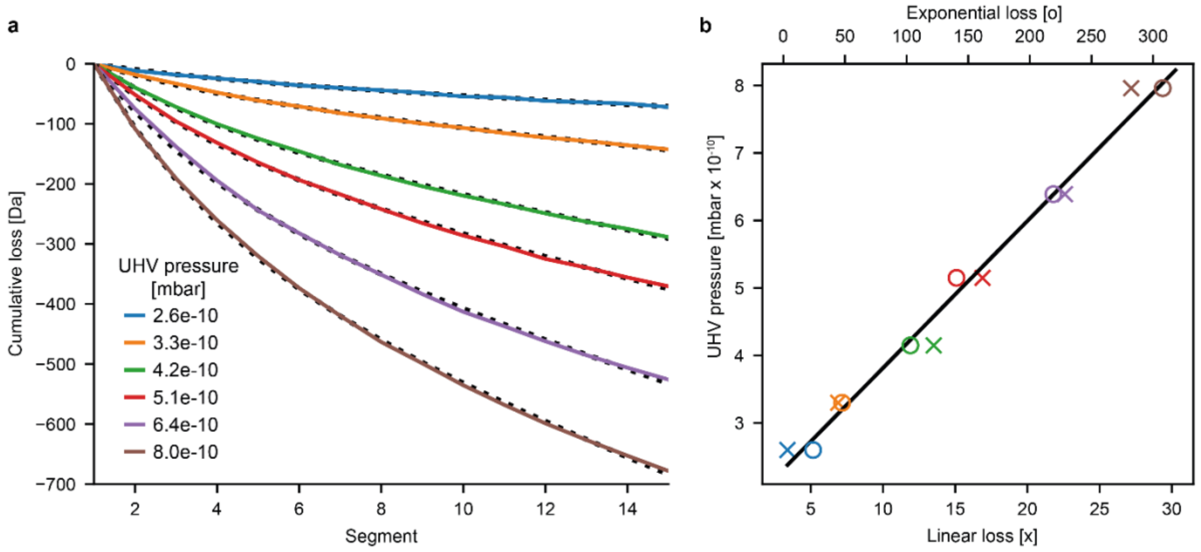

Supplemental Figure 3: **a**, Average total neutral loss of individual ions, with respect to the first segment, plotted for a wide range of pressures. The experimental cumulative neutral loss can be described accurately with the function: *Cumulative_loss*(*Segment*) = *Exponential_loss* * $e^{-0.3*Segment}$+ *Linear_loss* * *Segment.* The resulting simulations are shown as dotted lines with r^2-values in the range of 0.998-0.9998. **b**, The corresponding values for the exponential neutral loss and linear neutral loss are plotted against the UHV pressure and show a nice linear correlation.

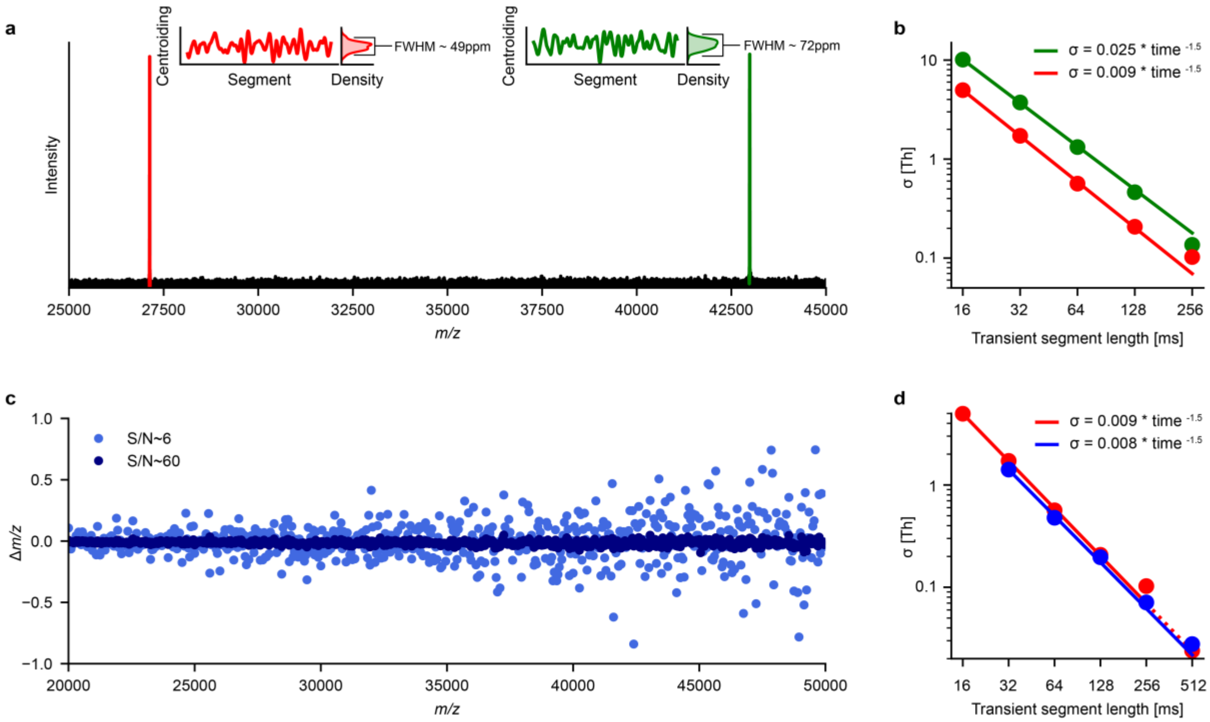

Supplemental Figure 4: a, Experimental spectrum of a viral particle ion at 42980 Th with a "spiked in" synthetic species at 27130 Th. The temporal instabilities of the experimental (green) and the synthetic (red) peaks as well as the overall distribution of the observed centroids are shown as insets. **b,** The observed *m/z* stability as a function of the (sub-) transient duration. The *σ*'s of the overall distribution of the observed centroids of the experimental (green) and the synthetic (red) centroids were fitted to an exponential function. **c** The observed *m/z* uncertainty as a function of signal to noise ratio and the *m/z* values. The results of the shotgun type of computational experiments investigating the spread of the observed values (in terms of *σ*) *m/z* across the 2 kTh-5 kTh range as a function of S/N (6 to 60). **d**, The comparison of the observed *m/z* value spread in "Temporal Stability" vs "In Silico Shotgun" experiments. The observed *σ*'s of the *m/z* values of the synthetic peak at 27130 Th in the temporal evolution of the resonance frequency analysis experiments (red) and shotgun experiments (mass range is limited to 27000 Th to 27200 Th in blue) as a function of the time domain signal length. The *σ* value for the transient duration of 0.5 seconds (shown in red) is extrapolated (dotted line) using the observed relationship.

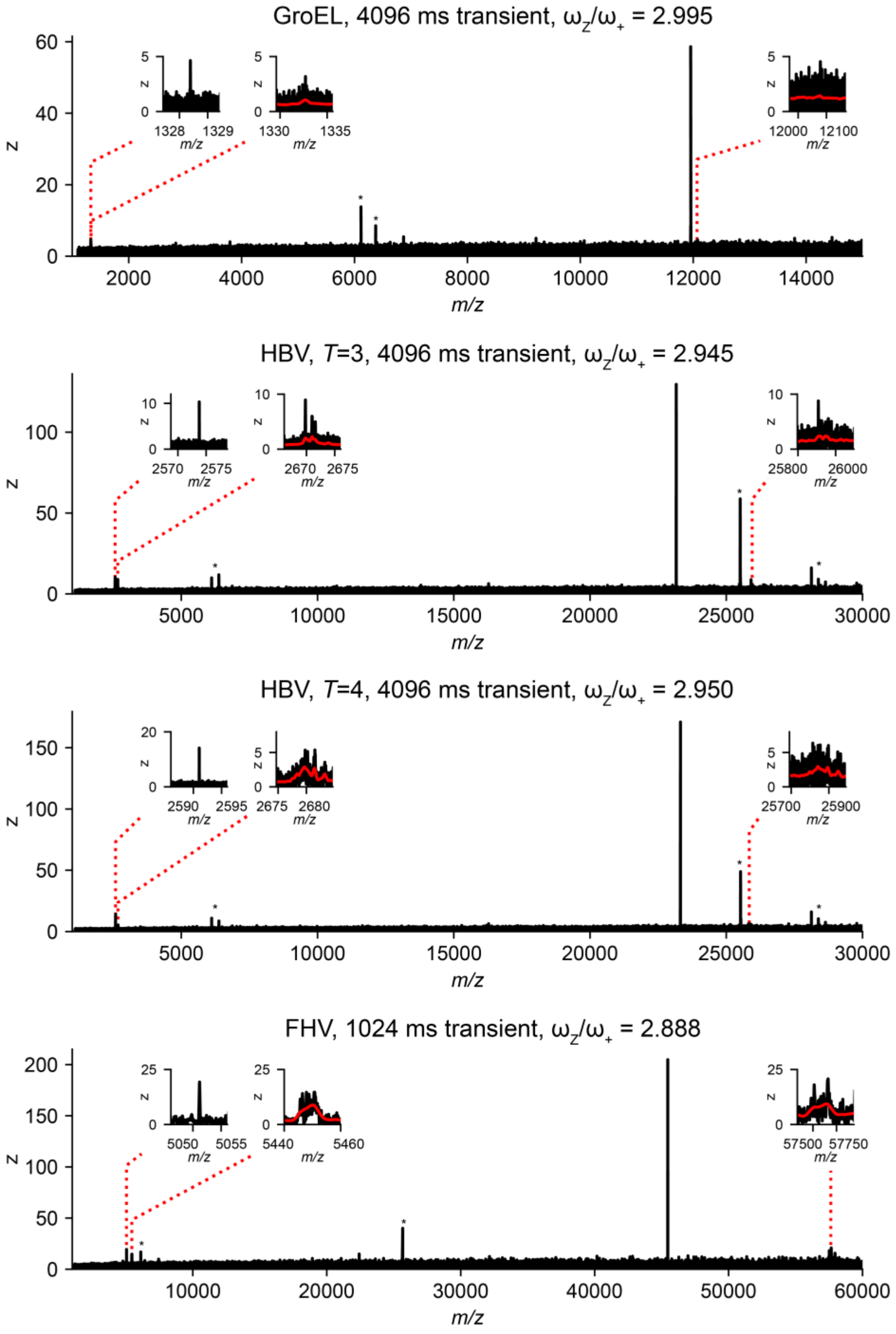

Supplemental Figure 5: Spectra of single ion occurrences for GroEL, HBV, *T*=3 and *T*=4 as well as FHV (top to bottom). Regions where the 3rd harmonic of ω_Z, the radial frequency modulation ω_+ and ω_-, occur are shown as insets (left to right). The modulations for ω_- are barely visible against the higher noise level at lower frequency and S/N for GroEL. This is contrasted by the highly charged FHV ion which modulations are clearly visible against the noise at transient duration of only 1s. We observed a continued trend to lower ω_Z/ω_+ for higher *m/z* values. Prominent electronic noise peaks are indicated with *.
