# Supplementary material for: Frequency chasing of individual megadalton ions in an Orbitrap analyser improves precision of analysis in single-molecule mass spectrometry
Source: Nat Chem. 2022 Mar 10;14(5):515–22. doi: 10.1038/s41557-022-00897-1 (PMC9068510; doi:10.1038/s41557-022-00897-1)
Supplement: Supplementary file 2 — Description of the code used with an example. [file 41557_2022_897_MOESM2_ESM.zip › Code_example/Example.html]

Example


In [1]:

```
#loading standard libraries
import matplotlib.pyplot as plt
import matplotlib.colors as c
import pandas as pd
import numpy as np
import pyteomics.mzxml #Might needs to be installed via "pip install pyteomics"

#Defining custom color map
custom_map = c.LinearSegmentedColormap.from_list("map", ["white", "red", "darkred"])
```

In [2]:

```
#class used for centroid tracing of segmented FT
class TraceIons:
    def __init__(self, path_input, select_scan=None):
        self.path_input = path_input
        self.select_scan = select_scan
        self.read_centroids()

    def read_centroids(self):
        self.data = pd.read_csv(self.path_input)
        if self.select_scan:
            self.data = self.data[self.data["scan"].between(self.select_scan[0], self.select_scan[1])]

    def trace_ions(self):
        filtered = []
        for scan, df in self.data.groupby("scan"):
            #plt.plot(df["m/z"], df["segment"], ".")
            for segment, df2 in df.groupby("segment"):
                try:
                    bol = list(df2["m/z"].diff()[1:] < -20)
                    bol = np.append(bol, [True]) & np.append([True], bol)
                    filtered.append(df2[bol])
                except:
                    pass

        filtered = pd.concat(filtered).reset_index()
        final = []
        c = 1
        for scan, df in filtered.groupby("scan"):
            first = df[df["segment"] == 1]
            later = df[df["segment"] > 1].copy()
            for idx, row in first.iterrows():
                later["diff"] = (later["m/z"] - row["m/z"]).abs()
                min_idx =later.groupby("segment")["diff"].idxmin()
                traced = filtered.iloc[min_idx]
                traced = traced.append(row).sort_values("segment")
                traced["ion_id"] = c
                if traced["m/z"].std() < 20:
                    final.append(traced)
                c+=1
        
            self.final = pd.concat(final)
```

In [3]:

```
#Read eFT centroids generated from .raw files using MSConvert

it=0.1
data = pyteomics.mzxml.read("./AaLS_eFT_centroids.mzxml")
scans = [i for i in data.iterfind("*") if "basePeakIntensity" in i]
mz = [s["m/z array"] for s in scans]
intensity = [s["intensity array"] for s in scans]
scans = [[int(s["num"])] * s["peaksCount"] for s in scans]
mz = np.concatenate(mz)
intensity = np.concatenate(intensity) * it
scans = np.concatenate(scans)

#put eFT centroids into datframe and plot 2D Histogram
AaLS_eFT = pd.DataFrame({"m/z" : mz, "Intensity" : intensity, "Scan" : scans})
AaLS_eFT["z"] = AaLS_eFT["Intensity"] / 12.521


#Plot eFT centroids illustrating peak splitting artifact

bins = (np.arange(19500, 23000, 20), np.arange(60,180,2))

fig, ax = plt.subplots(figsize=(4,4))
plt.hist2d(AaLS_eFT["m/z"], AaLS_eFT["z"], bins=bins, cmap=custom_map)
plt.xlabel("m/z")
plt.ylabel("z")
plt.title("eFT centroids")
```

Out[3]:

```
Text(0.5, 1.0, 'eFT centroids')
```

In [4]:

```
#Loading of segmetnd FT centroids and plotting of first segment.
#No peak splitting but very pooor charge resolution due to  used short transient segment

AaLS_untraced = TraceIons("./AaLS_segmentedFT.csv")


fig, ax = plt.subplots(figsize=(4,4))
plt.hist2d(AaLS_untraced.data[AaLS_untraced.data["segment"] == 1]["m/z"], AaLS_untraced.data[AaLS_untraced.data["segment"] == 1]["Intensity"]*175, bins=bins, cmap=custom_map)
plt.xlabel("m/z")
plt.ylabel("z")
plt.title("Single 128ms segment centroids")
```

Out[4]:

```
Text(0.5, 1.0, 'Single 128ms segment centroids')
```

In [5]:

```
# tracing ions and save as csv. This processing step can take 1-5 minutes

AaLS_untraced.trace_ions()
AaLS_untraced.final.to_csv("./AaLS_segmentedFT_traced.csv", index=False)
```

In [6]:

```
# loading of previously traced ions and filter wrong traced ions by applying threshold for standard deviation 
AaLS_traced_all = pd.read_csv("./AaLS_segmentedFT_traced.csv")
AaLS_traced_std = AaLS_traced_all.groupby("ion_id")[["m/z", "Intensity"]].std()
AaLS_traced = AaLS_traced_all.groupby("ion_id")[["m/z", "Intensity"]].mean()

fig, ax = plt.subplots(ncols=2, figsize=(10,4))


ax[0].hist(AaLS_traced_std["m/z"], bins=np.arange(0, 20, .5))
ax[1].hist(AaLS_traced_std["m/z"], bins=np.arange(0, 20, .5))

AaLS_traced = AaLS_traced[AaLS_traced_std["m/z"] < 4]
AaLS_traced_std = AaLS_traced_std[AaLS_traced_std["m/z"] < 4]
ax[0].hist(AaLS_traced_std["m/z"], bins=np.arange(0, 20, .5))
ax[1].hist(AaLS_traced_std["m/z"], bins=np.arange(0, 20, .5))


ax[0].set_xlim(0, 20)
ax[1].set_xlim(0, 20)
ax[0].set_ylim(0, 2500)
ax[1].set_ylim(0, 100)


ax[0].set_xlabel("STD(m/z)")
ax[1].set_xlabel("STD(m/z)")
ax[0].set_ylabel("Count")
ax[1].set_ylabel("Count")
ax[0].set_title("STD of traced ions with 4 Th cutoff")
ax[1].set_title("Zoom in cutoff region")
```

Out[6]:

```
Text(0.5, 1.0, 'Zoom in cutoff region')
```

In [7]:

```
# Comparison of 2d histrograms for eFT centroids vs single segment vs traced segments

fig, ax = plt.subplots(ncols=3, figsize=(15,4))
ax[0].hist2d(AaLS_eFT["m/z"], AaLS_eFT["z"], bins=bins, cmap=custom_map)

ax[1].hist2d(AaLS_untraced.data[AaLS_untraced.data["segment"] == 1]["m/z"], AaLS_untraced.data[AaLS_untraced.data["segment"] == 1]["Intensity"]*175, bins=bins, cmap=custom_map)

ax[2].hist2d(AaLS_traced["m/z"], AaLS_traced["Intensity"]*175, bins=bins, cmap=custom_map)

ax[0].set_title("eFT")
ax[1].set_title("Segmented FT, 1 segment")
ax[2].set_title("Segmented FT, 15 segments traced")

ax[0].set_ylabel("z")
ax[1].set_ylabel("z")
ax[2].set_ylabel("z")

ax[0].set_xlabel("m/z")
ax[1].set_xlabel("m/z")
ax[2].set_xlabel("m/z")
```

Out[7]:

```
Text(0.5, 0, 'm/z')
```
